# Atmospheric Plasma Lingual Frenectomy Followed by Post Operative Tongue Exercises: A Case Series

**DOI:** 10.3390/children10010105

**Published:** 2023-01-04

**Authors:** Antonio Scarano, Rosanna Di Giulio, Sergio Alexandre Gehrke, Gianluca Tagariello, Francesco Romano, Felice Lorusso

**Affiliations:** 1 Department of Innovative Technologies in Medicine and Dentistry, University of Chieti–Pescara, 66100 Chieti, Italy; 2 Department of Research, Bioface/PgO/UCAM, Calle Cuareim 1483, Montevideo 11100, Uruguay; 3 Via Pietro Calamandrei, 4, 74121 Taranto, Italy; 4 Pzza Castelnuovo 26/A, 90141 Palermo, Italy

**Keywords:** tongue-tie, frenotomy, atmospheric plasma, lingual frenulum, ankyloglossia

## Abstract

The lingual frenulum is a submucosal component significantly involved in the mobility of the tongue. In the case of short lingual frenulum, different surgical approaches have been proposed. Atmospheric plasma is a thermal technique of vaporization or sublimation of the superficial tissues, without going deep, and the resulting fine carbonized layer avoids bleeding. The aim of the present investigation was to evaluate the effectiveness of atmospheric plasma (voltaic arc dermabrasion) for the frenectomy of a short lingual frenulum. A total of 30 patients with an age range between 6–11 years old and a class III/IV Kotlow’s ankyloglossia classification were included in the study. The Kotlow’s free-tongue, maximal interincisal mouth opening (MIO, mm) and interincisal mouth opening with tongue tip to maxillary incisive papillae at roof of mouth (MOTTIP, mm) were calculated at the baseline, immediately postoperatively (T0), at one week (T1), one month (T2) and two months (T3). A significant increase of Kotlow’s measurements, MOTTIP and MIO were detected when comparing the baseline and the T0 (*p* < 0.05). No significant difference was detected between the T0, T1 and T2 (*p* > 0.05). The atmospheric plasma demonstrated a very minimal invasive approach for frenectomy, without important or fibrotic complications and with very low recurrence rates.

## 1. Introduction

The lingual frenulum represents the midline mucosal component that develops between the tongue lower surface and the mouth floor [1]. The structure is characterized by a submucosal component producing a complete division of the middle connective component structure, and it is involved in the mobility of the tongue [2,3]. In the literature, a discrete inter-individual variability regarding the position was reported, with the frenulum’s attachment length on the tongue’s ventral surface; these properties have been considered the formal parameters of Kotlow’s grading system for a tongue-tie diagnosis [4]. In fact, Kotlow’s grading classification is based on the lingual frenulum’s visual appearance in order to classify it as a “tongue tie”. The “anterior tongue tie” is defined as a frenulum with closer attachment to the tongue tip, while the “posterior tongue-tie” is associated to a frenulum characterized by a lower ventral tongue position; a “submucosal” frenulum develops and is not at all visible, associated to “tension” or “restriction” at the level of the mouth floor [5,6,7,8]. As the categories of these grading systems encompass the full range of possible variation in frenulum appearance, they allow any frenulum to be categorized as a “tongue tie” and to therefore be labelled as “abnormal.” This creates a dilemma regarding when a lingual frenulum’s appearance can be considered normal, and potentially drives an international trend for an increasing rate of diagnosis of ankyloglossia, as reported in Canada, Unites States of America and Australia [9,10,11]. These authors all voice concerns regarding the potential for overdiagnosis and a need for improved diagnostic criteria to avoid unnecessary surgery. The tongue-tie or ankyloglossia condition prevents the tongue from protruding beyond the lower incisor teeth because of a short frenulum linguae, often containing scar tissue [12]. This condition limits lingual movement and makes breastfeeding difficult [13] it can cause orthodontic disorders [14], gingival recession and related periodontal implications [15]. A short lingual frenulum limits upward movement, such as in the course of deglutition, when the tongue pushes anteriorly instead of upward to the hard palate, reducing palatal width [6]. Management of a short lingual frenulum is generally surgical by frenotomy or frenectomy and frenuloplasty. Frenectomy provides complete excision of the whole frenum. Many techniques have been used for frenectomy, such us different types of lasers, cold blade and electrosurgery. Atmospheric plasma is an innovative technique based on a plasma arc. This device is able to produce a vaporizing/sublimation of the soft tissue surface through a plasma arc. This non-invasive technique has proved its effectiveness for many different clinical procedures: i.e., benign lesion removal from the face, regenerative endodontic procedures and aesthetic medicine [16,17,18]. In this study, the authors evaluated the clinical efficacy of atmospheric plasma (voltaic arc dermabrasion) for the treatment of short lingual frenulum. The null hypothesis was that the atmospheric plasma produced no improvements of MOTTIP, MIO and Kotlow’s parameters at the study timepoints.

## 2. Materials and Methods

### 2.1. Study Design

**Figure 1:**
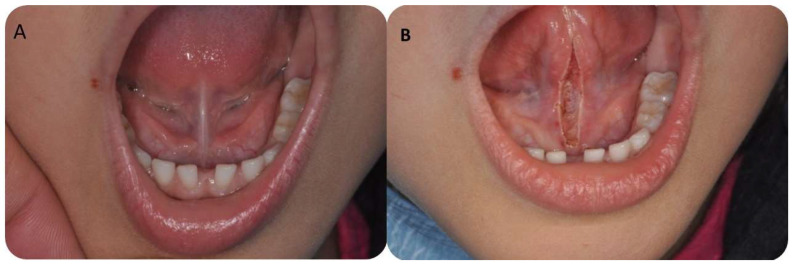
(**A**) Clinical aspect before atmospheric plasma treatment. (**B**) After atmospheric plasma treatment.

**Figure 2:**
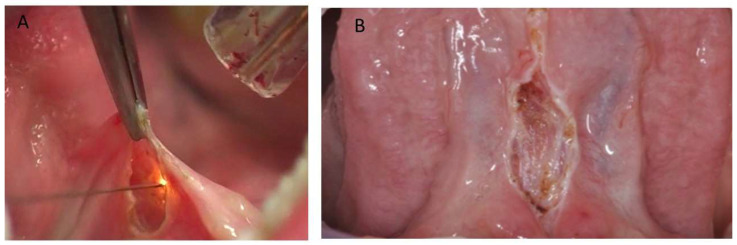
(**A**) During plasma jet. (**B**) Clinical aspect after frenectomy with atmospheric plasma jet; no bleeding was observed.

**Figure 3:**
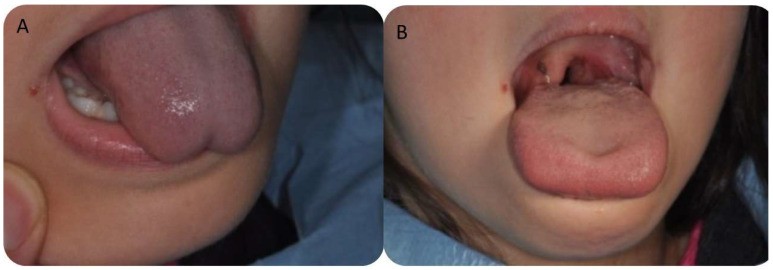
(**A**) Clinical aspect of the tongue is extremely limited before the treatment. (**B**) The mobility is visibly improved immediately post-treatment.

This retrospective study was conducted from November 2016 to October 2019. The investigation was conducted in accordance with the ethical principles of the European Union rules on good clinical practice, according to the declaration of Helsinki and the additional requirements of Italian law. The authors treated 30 children (16 female and 14 male) aged from 6 to 11 years (with an average age of 8.8 years) presenting ankyloglossia classified as class III or IV, according to Kotlow’s classification, with the atmospheric plasma technique. All children received a complete oral and lingual examination at the initial visit and standardized photographs were taken (Figure 1, Figure 2 and Figure 3). Post-operative visits were at one week, one month and two months. At each visit, careful attention was paid to lingual movement, mouth opening and lingual scarring. 

### 2.2. Clinical Measurements

Measurements were taken before and after the procedure of maximal interincisal mouth opening (MIO, mm), interincisal mouth opening with tongue tip to maxillary incisive papillae at roof of mouth (MOTTIP, mm), Kotlow’s free-tongue measurement (length from base of tongue insertion of the lingual frenulum to the tip) and presence of severe clinically apparent ankyloglossia, using Kotlow’s structural guidelines [4]. During the measurements the patients had their eyes focused on a point in the distance at eye level with their head positioned in a natural posture. Each measurement was achieved by the Quick Tongue Tie Assessment Tool^®^ (QTT) [19].

### 2.3. Surgical Procedure

The lingual frenulum was removed using atmospheric plasma also known as voltaic arc dermabrasion (VAD, Europe Medical s.r.l., Montesilvano (PE), Italy). It is known that a local administration of an anesthetic spray (lidocaine spray Ogna S.p.A., Milan, Italy) or infiltration of a few drops of Articaine (Pierrel S.p.A., Milan, Italy) is enough to avoid suffering any pain during this treatment. One incision was by the plasma device cutting through the lower aspects of the frenulum, and thus a linear tissue was completely removed (Figure 1B and Figure 2). During the session, the frenulum was treated once with a series of spots using a 50 kHz high voltage alternating current power supply (3 kV, 2 mA) with 2 W. No suture was applied. Photographs taken before and after the treatment were used by a joint examiner to evaluate the outcome of the study. All the procedures were performed by the same operator for all the patients at the Oral Surgery Unit of the University of Chieti–Pescara (Italy). During the procedure the tongue was gently pulled and lifted towards the palate in order to prevent deformation and misshaping. After the atmospheric procedure special instructions were given: not to eat spicy food and not to consume salad with salt and vinegar. No anti-inflammatory or antibiotic therapy was required, but rinses with chlorhexidine mouthwash (3/4 a day) for a week were prescribed. Immediately after the procedure the patient was referred to a speech therapy specialist and returned to their normal routine immediately, with speech therapy through functional exercises [20]. In detail, Ferres-Amat summarized the following post-intervention protocol [20]:

- 1st week before intervention: lingual exercises (one sequence 15 times/day)
- 24 h after surgery: lingual exercises (two sequence 15 times/day)
- 48 h after surgery: lingual exercises (three sequence 15 times/day)
- 72 h after surgery: execution control
- next 15 days: lingual exercises (three sequence 15 times/day)
- 15 days after: execution control
- next 30 days: lingual exercises (one sequence 15 times/day)
- 45 days after: execution control

### 2.4. Statistical Methodologies

The Kotlow’s free-tongue measurement, MIO and MOTTIP were recorded before and after the procedure (T0), at one week (T1), one month (T2) and two months (T3) using a special designed case report form. The study data were statistically analyzed through the statistical software package GraphPad 8 (Prism, San Diego, CA USA). The normal distribution of the study data was assessed by the Kolmogorov–Smirnov test followed by Pearson’s Correlation test. The One-Way ANOVA Tukey’s was applied for a multiple comparison test considering a level of significance for *p* < 0.05.

## 3. Results

**Figure 4:**
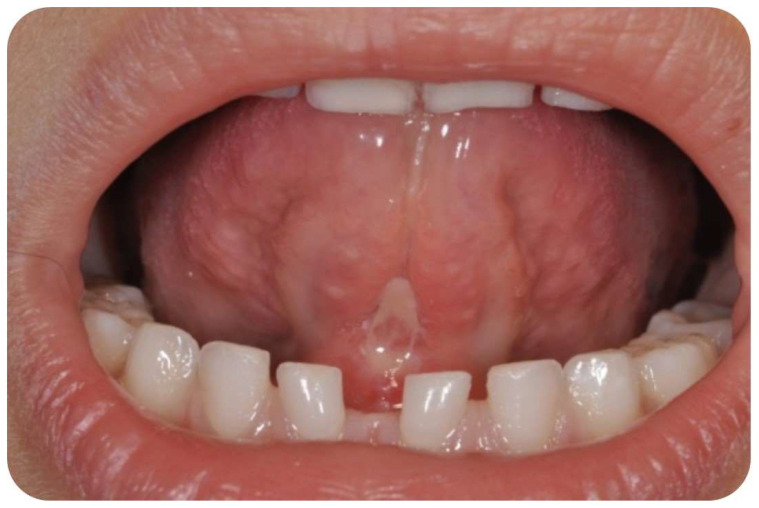
No bleeding or edema and good soft tissue healing after seven days.

No discomfort was reported at the conclusion of the atmospheric plasma treatments. Immediately after the atmospheric procedure, a clear perception of the ease in tongue movement and improvement in the level of the mobility of the tongue were observed. No edema or pain were referred by any of the patients (Figure 4). After surgery, relapse occurred in three cases and a second surgery was required. In these patients a local restriction was observed indicating inadequate wound contraction, stiffening and fibrotic healing, or the tissues were re-coaptated causing a lack of lingual movement as previously achieved after the surgery. A mild bleeding was found more frequently during and immediately after procedure, but no bleeding was observed after five/ten minutes, and in no case was it necessary to perform antihemorrhagic procedures. After one month and two months an improvement in tongue mobility was confirmed.

### 3.1. Data Evidence

The descriptive statistics and Pearson’s correlation test output are presented in Table 1 and Table 2. The Pearson coefficient (r) produced values ranged between −1 and 1, where:

- 0 < r_xy_ < 0.3 weak correlation (positive/negative)
- 0.3 < r_xy_ < 0.7 moderate correlation (positive/negative)
- 0.7 < r_xy_ < 1 strong correlation (positive/negative)

For almost all timepoint comparisons, the Kotlow’s, MOTTIP and MIO measurements showed a weak correlation (r_xy_ < 0.03) (Table 1 and Table 2).

### 3.2. Clinical Measurements

The average Kotlow baseline and postoperative measurement were, respectively, 17.2 ± 2.10 (95% CI: 16.4–18.0) and 27.1 ± 4.10 (95% CI: 16.4–18.0) showing a significant increase of the free-tongue measurement (*p* < 0.05). At the same time, a significant increase of the MOTTIP and MIO were detected between the baseline and immediately after the procedure (T_0_) (*p* < 0.05). The mean MOTTIP at the baseline and T0 were, respectively, 17.7 ± 4.63 (95% CI: 15.9–19.4) and 23.0 ± 3.63 (95% CI: 21.6–24.3). The average MIO before and after treatment were 30.0 ± 5.50 (95% CI: 27.9–32.0) and 42.1 ± 5.00 (95% CI: 40.2–43.9) (Table 1).

### 3.3. Postoperative Findings

The post hoc comparison between the study timepoints has been summarized in Table 3.

**Figure 5:**
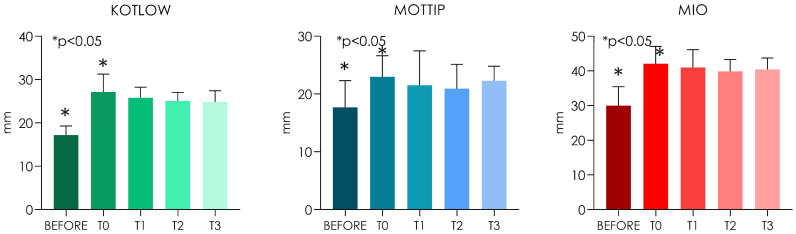
Chart of the Kotlow’s free-tongue measurement, MOTTIP and MIO at T_0_, T_1_, T_2_ and T_3_ (One-Way ANOVA Tukey’s post hoc).

No significant differences were detected between T_1_, T_2_ and T_3_ regarding Kotlow’s free-tongue measurement, MOTTIP and MIO (*p* < 0.05) (Figure 5). The mean Kotlow’s measurements at T1 and T_2_ were, respectively, 25.8 ± 2.48 (95% CI:24.8–26.7) and 25.1 ± 1.99 (95% CI: 24.3-25.8). At two months (T_3_), The mean Kotlow’s free tongue was 24.9 ± 2.55 (95% CI: 23.9–25.8) (Figure 1). The average MOTTIP at T_1_ and T_2_ were 21.5 ± 5.96 (95% CI: 19.3–23.7) and 20.9 ± 4.21 (95% CI: 19.4–22.5). After two months (T_3_) from the treatment, the MOTTIP average was 22.3 ± 2.53 (95% CI: 21.3–23.2) (Figure 5). At T_1_ and T_2_, the maximal interincisal mouth opening (MIO) means were, respectively, 41.0 ± 5.14 (95% CI: 39.0–42.9) and 39.9 ± 3.42 (95% CI: 38.6–41.1). At T_3_, the MIO values were 40.4 ± 3.35 (95% CI: 39.1–41.6) (Figure 5).

## 4. Discussion

In the present study, a case series of patients diagnosed with tongue-tie and treated with atmospheric plasma are presented. Due to the novelty of the procedure, the lack of comparative studies, the applied experimental methodology and its limits, the present investigation has been conducted in form of a pilot study. One of the main objectives of the study was also to determine the effect size of the therapies and adequate homogeneous population sampling for further clinical controlled trials. The findings show an improvement of MIO, MOTTIP and Kotlow’s free-tongue, and the null-hypothesis was rejected. The speech assessment is a very arbitrary parameter that could be subject to a potential measurement bias. For this reason, it was not considered an objective of this pilot study. As expected, the maximum effect of the lingual frenectomy with atmospheric plasma is visible in the immediate perioperative period with a significant increase of Kotlow’s free-tongue of about 10 mm, with an appreciable stability at all postoperative timepoints up to two months from the treatment. Similar effectiveness was detected for the other complementary clinical parameters, MIO and MOTTIP. According to Yoon et al., MIO could be mildly correlated to age and height, while MOTTIP is dependent on MIO. The same authors reported that Kotlow’s measurement represents an independent assessment of free-tongue length and tongue mobility [19]. So, the outcome confirms that lingual frenectomy with atmospheric plasma leads to an improvement in tongue mobility. The reports on lingual frenectomy failure and complications are severely lacking; the major ones are represented by breastfeeding and poor feeding evidence and speech impediment [21]. 

Limitations to the present study model are clearly correlated to the reduced population sampling, the non-blind protocol and single operator measurements. In addition, other hypothesized limitations could be associated to no selection regarding ethnic groups and age homogeneity. Atmospheric plasma is generated by an electric discharge which causes ionization, excitation or dissociation of air molecules leading to creation of vaporization or sublimation of the superficial tissues, without going deep, and the resulting fine carbonized layer disappears after two weeks. This device is widely used in aesthetic medicine for eradication of a skin lesions or for the treatment of skin wrinkles [22,23]. The use of this device in aesthetic medicine means that it does not induce thermal damage, such as to the generation of scars. Many techniques have been used for frenectomy such us a cold-blade, laser [24] and electrosurgery. Cold-blade scalpel promotes greater bleeding compared with electrocautery and laser or tools. Frenectomy is a removal of the fibers that cause the limitation of tongue movements, which is fundamental. 

The lingual frenulum is characterized by a midline connective component that is associated by a central band surrounded by a fascial layer that rises across the mouth floor. This layer attaches circumferentially around the inner mandible surface, with the fascia “flaring” into horizontal components to give an increased vertical attachment that continues with the mandible periosteum. The superior component height of the attachment layer is able to produce the upper limit part with the mouth floor, in close relationship with the oral mucosa separated with the gingiva. The fibers within the mouth fascial layers floor develop centrally, following the close contours of the mucosa, merging with the dense submucosal connective tissue at the level of the tongue ventral surface. In a few cases, the frenectomy needs additional treatment, because postoperative fibrotic scarring generated by the fibrotic tissue can also be formed by the fusion of the mucosa with the floor oral fascia, with the loss of tongue mobility and the occurrence of relapse. Another cause for requiring additional treatment is the re-coaptation of tissues during the healing period. The high temperature emitted at the surgical site from the diode laser accelerates the formation of fibrotic scar tissue [25]. When there is formation of fibrotic tissue, the floor-of-the-mouth (FOM) fascia—which creates a diaphragm-like structure that suspends the tongue within the arc of the mandible—is compromised, probably by fusion of the oral mucosa with the floor mouth oral fascia. With all anterior tongue movements, the central attachment of the fascia to the ventral tongue surface creates passive movement of the FOM fascia, together with the overlying oral mucosa [26]. In fact, the lingual frenulum histologically is a dynamic structure formed by a midline fold of the FOM fascia together with the overlying FOM mucosa, so mobilization of the mucosal and fascial layers appear to impact frenulum morphology [26]. The plasma discharge causes rapid heating of the skin, with limited skin ablation, with contraction of collagen around the vessel and minimal collateral thermal damage [27]. 

## 5. Conclusions

This novel research has shown that the atmospheric plasma is a minimally invasive method, successfully used for frenectomy, without important or fibrotic complications and with very low recurrence rates. Future clinical studies with a greater number of patients should be carried out to compare lingual frenectomy surgery with other methods including minimally invasive ones.

## Figures and Tables

**Table 1:** Descriptive statistic according to Kotlow’s free-tongue measurement, MOTTIP and MIO. (mean; standard deviation, 95% CI).

	Kotlow					MOTTIP					MIO				
	BEFORE	T0	T1	T2	T3	BEFORE	T0	T1	T2	T3	BEFORE	T0	T1	T2	T3
Mean	17.2	27.1	25.8	25.1	24.9	17.7	23.0	21.5	20.9	22.3	30.0	42.1	41.0	39.9	40.4
Std. Deviation	2.10	4.10	2.48	1.99	2.55	4.63	3.63	5.96	4.21	2.53	5.50	5.00	5.14	3.42	3.35
Lower 95% CI	16.4	25.6	24.8	24.3	23.9	15.9	21.6	19.3	19.4	21.3	27.9	40.2	39.0	38.6	39.1
Upper 95% CI	18.0	28.7	26.7	25.8	25.8	19.4	24.3	23.7	22.5	23.2	32.0	43.9	42.9	41.1	41.6

**Table 2:** Pearson’s coefficient (r) table of Kotlow’s free-tongue measurement, MOTTIP and MIO.

		Kotlow					MOTTIP					MIO				
		BEFORE	T0	T1	T2	T3	BEFORE	T0	T1	T2	T3	BEFORE	T0	T1	T2	T3
Kotlow	BEFORE	-	-	-	-	-	0.054	−0.045	−0.203	−0.055	−0.011	−0.345	0.245	0.051	−0.182	0.106
	T0	-	-	-	-	-	−0.2779	0.109	0.333	−0.007	−0.176	0.042	−0.065	0.032	0.23	0.099
	T1	-	-	-	-	-	0.159	0.048	−0.329	0.013	0.125	−0.094	0.129	0.149	−0.06	−0.222
	T2	-	-	-	-	-	−0.143	−0.036	−0.074	0.076	−0.381	0.157	0.034	0.335	0.102	−0.565
	T3	-	-	-	-	-	−0.31	0.387	−0.122	0.219	−0.321	0.198	−0.363	−0.176	0.248	0.289
MOTTIP	BEFORE	0.053	−0.278	0.159	−0.143	−0.31	-	-	-	-	-	−0.164	0.0511	0.123	−0.211	0.113
	T0	−0.045	0.101	0.048	−0.036	0.387	-	-	-	-	-	−0.013	−0.229	0.052	−0.123	0.036
	T1	−0.203	0.333	−0.329	−0.074	−0.122	-	-	-	-	-	−0.191	0.099	−0.074	0.345	0.071
	T2	−0.055	−0.007	0.013	0.076	0.219	-	-	-	-	-	−0.044	−0.167	−0.104	−0.019	0.203
	T3	−0.011	−0.176	0.125	−0.381	−0.322	-	-	-	-	-	−0.196	0.360131	−0.311	−0.301	0.037
MIO	BEFORE	−0.345	0.042	−0.095	0.157	0.199	−0.164	−0.013	−0.191	−0.044	−0.196	-	-	-	-	-
	T0	0.244	−0.064	0.129	0.034	−0.362	0.051	−0.229	0.098	−0.167	0.36	-	-	-	-	-
	T1	0.051	0.031	0.149	0.335	−0.175	0.123	0.051	−0.073	−0.104	−0.31	-	-	-	-	-
	T2	−0.182	0.23	−0.06	0.103	0.248	−0.211	−0.123	0.345	−0.019	−0.301	-	-	-	-	-
	T3	0.107	0.099	−0.222	−0.566	0.289	0.113	0.036	0.071	0.203	0.037	-	-	-	-	-

**Table 3:** Summary of the One-Way ANOVA Tukey’s multiple comparisons post hoc test.

Kotlow ANOVA Tukey’s Multiple Comparisons	Mean Diff,	95.00% CI	Adjusted *p* Value	
BASELINE vs. T0	−9.965	−11.93 to −8.004	<0.0001	A–B
BASELINE vs. T1	−8.589	−10.55 to −6.628	<0.0001	A–C
BASELINE vs. T2	−7.875	−9.836 to −5.913	<0.0001	A–D
BASELINE vs. T3	−7.68	−9.641 to −5.719	<0.0001	A–E
T0 vs. T1	1.376	−0.5854 to 3.337	0.3022	B–C
T0 vs. T2	2.091	0.1294 to 4.052	0.0304	B–D
T0 vs. T3	2.285	0.3239 to 4.246	0.0136	B–E
T1 vs. T2	0.7148	−1.246 to 2.676	0.8519	C–D
T1 vs. T3	0.9093	−1.052 to 2.870	0.7034	C–E
T2 vs. T3	0.1945	−1.767 to 2.156	0.9988	D–E
MOTTIP Tukey’s multiple comparisons test	Mean Diff,	95.00% CI of diff,	Adjusted *p* Value	
BASELINE vs. T0	−5.306	−8.401 to −2.210	<0.0001	A–B
BASELINE vs. T1	−3.824	−6.920 to −0.7288	0.0073	A–C
BASELINE vs. T2	−3.261	−6.357 to −0.1655	0.0335	A–D
BASELINE vs. T3	−4.609	−7.704 to −1.513	0.0006	A–E
T0 vs. T1	1.481	−1.614 to 4.577	0.6782	B–C
T0 vs. T2	2.044	−1.051 to 5.140	0.3636	B–D
T0 vs. T3	0.6966	−2.399 to 3.792	0.9714	B–E
T1 vs. T2	0.5633	−2.532 to 3.659	0.987	C–D
T1 vs. T3	−0.7845	−3.880 to 2.311	0.9562	C–E
T2 vs. T3	−1.348	−4.443 to 1.748	0.7498	D–E
MIO Tukey’s multiple comparisons test	Mean Diff,	95,00% CI of diff,	Adjusted *p* Value	
BASELINE vs. T0	−12.12	−15.38 to −8.858	<0.0001	A–B
BASELINE vs. T1	−11.01	−14.27 to −7.745	<0.0001	A–C
BASELINE vs. T2	−9.899	−13.16 to −6.638	<0.0001	A–D
BASELINE vs. T3	−10.43	−13.69 to −7.171	<0.0001	A–E
T0 vs. T1	1.113	−2.148 to 4.374	0.8796	B–C
T0 vs. T2	2.221	−1.040 to 5.482	0.3322	B–D
T0 vs. T3	1.687	−1.574 to 4.948	0.6101	B–E
T1 vs. T2	1.108	−2.154 to 4.369	0.8815	C–D
T1 vs. T3	0.5738	−2.687 to 3.835	0.9885	C–E
T2 vs. T3	−0.5338	−3.795 to 2.727	0.9913	D–E

## Data Availability

All experimental data to support the findings of this study are available contacting the corresponding author upon request.
